# Disruption of the Non-Canonical WNT Pathway in Lung Squamous Cell Carcinoma

**Published:** 2008-04-01

**Authors:** Eric H.L. Lee, Raj Chari, Andy Lam, Raymond T. Ng, John Yee, John English, Kenneth G. Evans, Calum MacAulay, Stephen Lam, Wan L. Lam

**Affiliations:** 1 Department of Cancer Genetics and Developmental Biology, BC Cancer Agency Research Centre, Vancouver, BC, Canada; 2 Department of Computer Science, University of British Columbia, Vancouver, BC, Canada; 3 Department of Surgery, Vancouver General Hospital, Vancouver, BC, Canada; 4 Department of Pathology, Vancouver General Hospital, Vancouver, BC, Canada; 5 Department of Cancer Imaging, BC Cancer Agency Research Centre, Vancouver, BC, Canada

**Keywords:** WNT pathway, lung cancer, gene expression, NSCLC, non-canonical, squamous cell carcinoma

## Abstract

Disruptions of beta-catenin and the canonical Wnt pathway are well documented in cancer. However, little is known of the non-canonical branch of the Wnt pathway. In this study, we investigate the transcript level patterns of genes in the Wnt pathway in squamous cell lung cancer using reverse-transcriptase (RT)-PCR. It was found that over half of the samples examined exhibited dysregulated gene expression of multiple components of the non-canonical branch of the WNT pathway. In the cases where *beta catenin* (*CTNNB1*) was not over-expressed, we identified strong relationships of expression between *wingless-type MMTV integration site family member 5A* (*WNT5A*)/*frizzled homolog 2* (*FZD2*), *frizzled homolog 3* (*FZD3*)/*dishevelled 2* (*DVL2*), and *low density lipoprotein receptor-related protein 5* (*LRP5*)/*secreted frizzled-related protein 4* (*SFRP4*). This is one of the first studies to demonstrate expression of genes in the non-canonical pathway in normal lung tissue and its disruption in lung squamous cell carcinoma. These findings suggest that the non-canonical pathway may have a more prominent role in lung cancer than previously reported.

## Background

**Figure 1:**
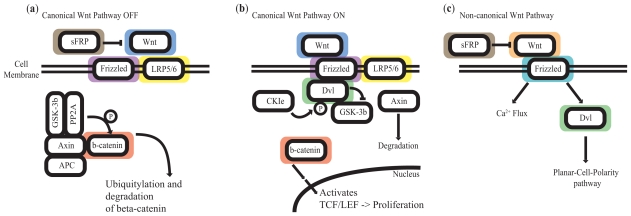
Schematic representation of the canonical and non-canonical Wnt pathways. sFRPs are inhibitors of both the canonical and non-canonical branches of the Wnt pathway. (**a**) Canonical Wnt pathway in its off state. (**b**) Canonical Wnt pathway in its on state. (**c**) Non-canonical Wnt pathway. Color halos represent genes that were used in this study. Grey: SFRP1, SFRP2, SFRP3, SFRP4, SFRP5; Blue: WNT1, WNT3A; Purple: FZD1; Yellow: LRP5, LRP6; Red: CTNNB1; Orange: WNT5A, WNT11; Teal: FZD2, FZD3, FZD6; Green: DVL2.

The Wnt pathway is integral to developmental biology. The canonical pathway determines β-catenin stability and influences the transcription of *TCF/LEF* target genes (Clevers, 2006). In the absence of Wnt ligands binding to frizzled receptors, the canonical Wnt pathway is turned off leading to the eventual degradation of β-catenin (Fig. 1A). Conversely, the binding of Wnt ligands promotes the formation of a tertiary complex between Wnt, Frizzled and LRP5/6, allowing β-catenin to shuttle into the nucleus and bind to TCF/LEF proteins, thus activating target gene transcription (Fig. 1B). The non-canonical pathway is β-catenin-independent and controls cell movements during morphogenesis. It is further subdivided into the Wnt/calcium pathway and the planar-cell-polarity (PCP) pathway (Fig. 1C) (Katoh, 2005; Veeman, Axelrod and Moon, 2003).

The canonical Wnt pathway plays a critical role during the development of the lung (Eberhart and Argani, 2001; Mazieres et al. 2005). In the adult lung, the canonical Wnt pathway contributes to bronchial epithelial regeneration (Steel et al.). However, little is known about the non-canonical pathway in the adult lung. Furthermore, disruption of the canonical pathway branch is well documented in cancer (Clevers, 2006; Ilyas, 2005), but the involvement of the non-canonical branch of the Wnt pathway in cancer is virtually unknown. Disruptions have been reported for many canonical pathway components; for example, mutations in axin and APC are common in colorectal and hepatocellular cancers (Aust et al. 2002; Taniguchi et al. 2002). The consequence of disrupting the Wnt pathway is the constitutive activation of target genes, such as *MYC*, *CCND1*, *VEGF*, each contributing to the hallmarks of cancer (Hanahan and Weinberg, 2000).

Lung cancer is a highly aggressive disease and is the leading cause of cancer deaths worldwide (Minna, Roth and Gazdar, 2002). Identification of genes and pathways disrupted in lung cancer will improve our understanding of this disease. Recent studies have implicated the disruption of upstream Wnt components in lung cancer. For example, *wingless-related MMTV integration site 1* (*WNT1*) and *wingless-related MMTV integration site 2* (*WNT2*) are overexpressed in non-small cell lung cancer (NSCLC) (He, B et al. 2004; You et al. 2004); loss of *wingless-related MMTV integration site family, member 7A* (*WNT7A*) contributes to the progression of lung cancer through its inability to induce E-cadherin (Ohira et al. 2003); and *DVL3* is reported to be overexpressed in NSCLC (Uematsu et al. 2003). However, disruption of downstream Wnt pathway components are not often reported in lung cancer (Shigemitsu et al. 2001; Ueda et al. 2001). Coordinated measurements of Wnt components expression will be necessary to define their involvement in lung cancer. In this study, we investigated the transcript level patterns of pathway components in normal lung tissue and lung squamous cell carcinoma (SCC) to determine if the expression of the non-canonical pathway is disrupted in lung cancer.

## Methods

### RNA isolation and cDNA synthesis

A total of 20 frozen squamous lung tumor with matched lung normal samples were obtained from St. Paul’s Hospital. Sections (10 μm) fixed in 70% ethanol were manually microdissected based on histopathologic evalution of hematoxylin and eosin stained sample sections by a lung pathologist. Dissected cells were homogenized in a guanidine thiocyanate lysis buffer and RNA was isolated using the RNeasy Mini Kit (Qiagen, Mississauga, ON, Canada). Matched normal lung tissue samples were homogenized in the presence of liquid nitrogen and RNA was extracted using Trizol reagent (Invitrogen, Burlington, ON, Canada). Purified total RNA (40 ng samples) was converted to cDNA using the Superscript II RNAse H reverse-transcriptase system (Invitrogen). Primer sequences and melting temperatures are described in Additional file 1. In addition, 10 frozen paired SCC samples were obtained for quantitative RT-PCR from Vancouver General Hospital. All samples for this study were collected with approval by the Review of Ethics Board of the Ministry of British Columbia.

### Gene expression analysis

Expression levels were determined by gene-specific PCR (Additional file 1) and the β-actin gene was used for normalization. cDNA samples obtained from tissues known to express the Wnt pathway were used as positive controls (Clontech human multiple tissue cDNA Panels 1 and 2, BD Biosciences Clontech, Mississauga, ON, Canada). Forty nanograms of RNA were converted to cDNA as described above and 1/20 of the cDNA from each sample was used. PCR cycle conditions were as follow: one cycle of 95 °C, 1 min; 30–35 cycles of 95 °C, 30 s; 55 °C, 30 s (for β*-actin*); 72 °C, 30 s; and a final 10 min extension at 72 °C. PCR products were resolved by polyacrylamide gel electrophoresis, imaged by SYBR green staining (Roche, Laval, PQ, Canada) on a Molecular Dynamics Storm Phosphoimager model 860, and quantified using ImageQuant software (Molecular Dynamics, Piscataway, NJ, U.S.A.). To verify the absence of genomic DNA contamination in the cDNA, a *ACTB* primer was designed to yield a 597 bp fragment for genomic DNA amplification product and a 400 bp fragment for cDNA amplification.

For quantitative PCR, TaqMan primers (primer IDs in parentheses) for *FZD3* (Hs00184043_m1), *DVL2* (Hs00182901_m1), and *CTNNB1* (Hs00170025_m1) were purchased from Applied Biosystems (Applied Biosystems, CA, U.S.A.). PCR was performed as recommended by Applied Bio-systems. All reactions were 25 μL in volume and performed in triplicate. To account for variations in template quantities, cycle threshold (Ct) values were normalized using the Ct values of *ACTB*. The efficiencies of all TaqMan primers were estimated using the raw data generated at each well as previously described (Liu and Saint, 2002; Weksberg et al. 2005).

### Statistical analysis of gene expression levels

Gene expression levels of Wnt pathway components were determined by calculating the signal intensity ratio between each gene of interest and *ACTB* was calculated for all lung samples. For the negative control, cDNA template was omitted in the reaction.

For the expression level comparison between tumor and normal tissue, the intensity ratio of each gene in tumor was divided by the corresponding intensity ratio in the matched normal tissue samples. Correlation coefficient analysis was performed using the Matlab Statistics Toolbox (The Mathworks, Natick, MA).

## Results and Discussion

Wnt pathway components representing the canonical and the non-canonical sub-paths were selected for expression analysis using RT-PCR in an effort to investigate the state of the pathways in normal lungs and their disruption in lung tumors. The genes representing the canonical pathway in this study include *WNT1, wingless-related MMTV integration site family, member 3A* (*WNT3A*)*, frizzled homolog* 1 (*FZD1*)*, low density lipoprotein receptor-related protein 5* (*LRP5*)*, density lipoprotein receptor-related protein 6* (*LRP6*), and *CTNNB1*. The non-canonical components were represented by *wingless-related MMTV integration site family, member 5A* (*WNT5A*)*, wingless-related MMTV integration site family, member 11* (*WNT11*)*, frizzled homolog 2* (*FZD2*)*, frizzled homolog 3* (*FZD3*), and *frizzled homolog 6* (*FZD6*) (Katoh, 2005; Pongracz and Stockley, 2006; Torres et al. 1996). In addition, representative members of the Dvl family and the sFRP family were also included in our analysis (Melkonyan et al. 1997; Schumann et al. 2000; Uematsu et al. 2003). It should be noted that the regulation of the wnt pathway is complex. Some of Wnt ligands may have the activation of both the non-canonical and canonical branches and as such, their effects are strongly dependent on the receptor.

**Figure 2:**
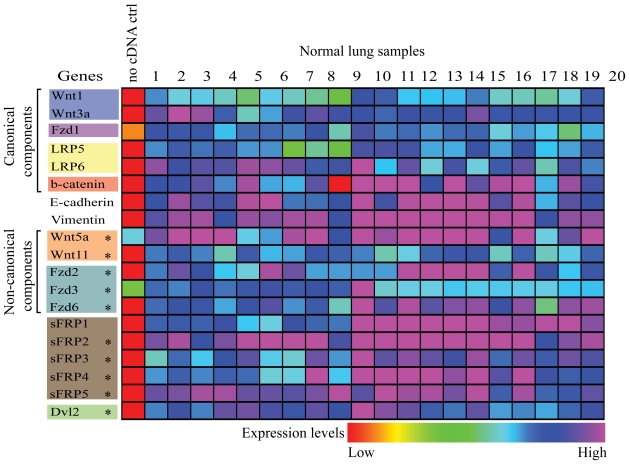
Expression profiles of 19 genes in 20 normal lung samples. Raw data was shifted by adding a constant to get rid of negative values. A trimmed mean was calculated (excluding the lower and upper 2% values) and a scaling factor was calculated as 500 divided by the trimmed mean. Each raw value was then multiplied by the scaling factor to create a new distribution centered at 500. The value displayed is the log_10_ of the scaled data. *represent expression of genes that have not been reported in normal lung in literature.

Expression profiles of the Wnt components in 20 normal lung samples are shown (Fig. 2). Analysis of the canonical Wnt pathway genes suggests their transcription in normal lung. Notably, the non-canonical Wnt components, *WNT5A*, *WNT11*, *FZD2*, *FZD3*, and *FZD6,* are also present in the normal lung. This is one of the first reports of non-canonical pathway expression in adult human non-malignant lung tissue (Pongracz and Stockley, 2006; Winn et al. 2005). In addition, *dishevelled 2, dsh homolog* (*DVL2*) *and* members of the *sFRP* family are also expressed in the normal lung (Fig. 2). Although the role of DVL2 is not entirely clear in humans, it has been shown to activate the PCP signaling pathway in a series of experiments involving HEK293T cell and *Xenopus* models (Habas, Kato and He, 2001). As for the sFRP family, not all members serve the same functions. For example, *sFRP2* enables the breast cancer cell line MCF-7 to resist TNF-induced apoptosis while *sFRP1* sensitizes the cells to TNF-induced apoptosis (Melkonyan et al. 1997). The gene expression data on normal lung tissue provide a baseline for comparison against those of NSCLC.

**Figure 3:**
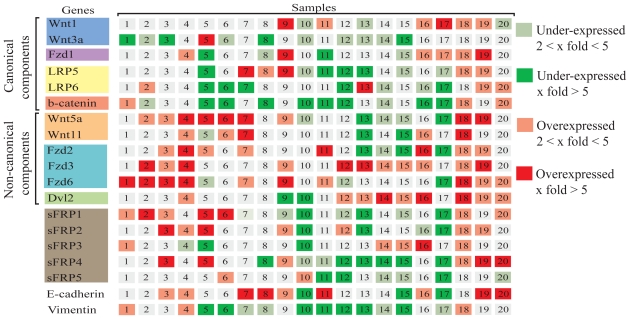
Expression data of the 19 genes in a pairwise comparison between lung tumors and their matched normals. Colored spots represent expression fold changes of genes by dividing tumor intensity ratio by the normal intensity ratio. Only 2 fold changes are displayed for the 20 tumor-normal pairs.

**Figure 4:**
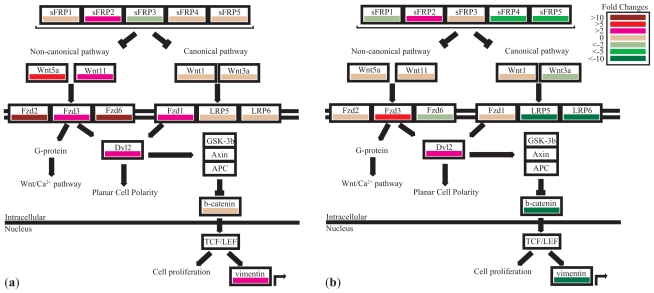
Comparison of pairwise (tumor versus matched normal) expression profiles between two patients. (**a**) Patient 7 show high level disruption of non-canonical WNT components and low to no change in expression of canonical WNT components. (**b**) Patient 10 shows high level disruption of canonical WNT components and low to no change in expression of non-canonical WNT components.

**Figure 5:**
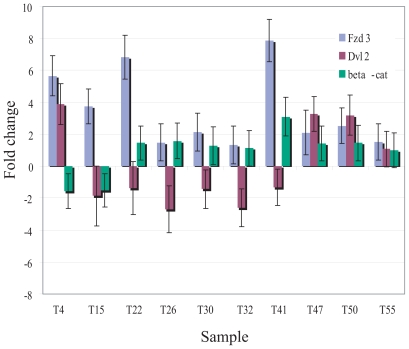
Differential expression of *FZD3*, *DVL2* and *CTNNB1* between 10 lung squamous cell carcinoma and matched normals (Samples T21–T30). Results are generated by real-time RT-PCR using TaqMan gene specific primers from Applied Biosystems.

To investigate which Wnt pathway components are disrupted in lung tumors, a pairwise comparison between tumour and matched normal lung samples was performed on the Wnt pathway genes (Fig. 3). A comparison of the components in the canonical and non-canonical pathway shows that the non-canonical pathway may be involved in a subset of tumor cases. For example, patient 4 (Fig. 4A) shows high level up-regulation of all non-canonical components while there is minimal disruption of the transcription levels of canonical components. In contrast, patient 12 (Fig. 4B) shows high level down-regulation of canonical components with minimal disruptions of the non-canonical components. In fact, the twenty samples have varying patterns of expression changes in the Wnt pathway components (Additional file 2). We only observed overexpression of *CTNNB1* in three out of 20 samples, and this observation held true in an independent set of ten cases by quantitative PCR (Fig. 5). This is not surprising as CTNNB1 activity is determined by protein stability and nuclear localization (Blache et al. 2004; He, TC et al. 1998; Korinek et al. 1997; Mann et al. 1999; Morin et al. 1997). However, it is remarkable that 11 out of 20 samples showed overexpression of multiple non-canonical components. These findings strongly suggest the involvement of the non-canonical pathway in lung SCC.

Based on the expression patterns of *CTNNB1*, it appears not all tumors solely involve the canonical pathway. We next investigated which particular non-canonical components are involved in the samples without *CTNNB1* overexpression. As some of the components affect both the canonical and non-canonical pathway, we selected only genes belonging to one or the other, namely those listed in Table 1. The expression of each gene was categorized as +1 for up-regulation, -1 for down-regulation, and 0 for unchanged, with a 2-fold expression difference deemed change. The genes were paired and a percentage was calculated for each pair of genes based on the number of times they showed the same category of expression. In other words, the percentage is an indication of how similar the expression changes are for a given set of genes. The table of gene comparisons with the corresponding percentages is shown in Table 1. Gene pairs that were less than 50% concordant in expression change were eliminated from further analysis. For the remaining gene pairs, a Spearman correlation was calculated. Eleven gene pairs showed statistically significant correlation with three gene pairs showing greater than 65% concordance: *LRP5 and secreted frizzled-related protein 4* (*SFRP4*), *WNT5A and FZD2*, and *FZD3 and DVL2*. We also investigated the frequency of discordant expression changes but, there were no gene pairs that were significantly related (data not shown).

The first pair of genes showing high concordance is *WNT5A and FZD2* (65%) with a correlation coefficient of 0.7 ( p < 0.01). *FZD2* and *WNT5A* are coordinately increased in 5 samples and decreased in 4 samples. The relationship between *WNT5A* and *FZD2* is novel in human lung but their association has been documented in other animal models. For example, previous studies in zebrafish models suggest that *Fzd2* induces intra-cellular release of Ca^2+^ via *Wnt5a* activation. The release of Ca^2+^ involves the activation of the phosphatidylinositol pathway in a G-protein-dependent manner (Kuhl et al. 2000; Sheldahl et al. 1999; Slusarski, Corces and Moon, 1997) which in turn activates CamKII and PKC. The implications of PKCs have been reported in various types of cancer. For example, human small cell lung cancer (SCLC) cells have shown to exhibit rapid growth due to over-expression of PKCɛ and similarly, breast cancer cells displayed an enhanced rate of proliferation due to PKCα transfection (Hofmann, 2004).

The next pair, the non-canonical components, *FZD3 and DVL2* are similar in 77% of the 17 tumor samples with a corresponding correlation coefficient of 0.6 ( p ≤ 0.01). We discovered that the expression levels of both *FZD3 and DVL2* are up-regulated in 7 out of 17 tumor samples and unchanged in 6 tumor samples where the expression of *CTNNB1* is down or unchanged. *FZD3* and *DVL2* have independently been reported to be involved in the non-canonical pathway. The patterns of expression of *FZD3* and *DVL2* do not seem to affect the expression levels of *CTNNB1*. Although the Dvl family has been shown to be able to activate the canonical and non-canonical pathway, *DVL2* alone does not display a high frequency of coordinate expression change with *CTNNB1* in this study. Likewise, *FZD3* alone does not seem to affect the expression of *CTNNB1* as well, which agrees with the majority of studies done on this gene. Quantitative RT-PCR was performed on *FZD3 and DVL2* on an independent set of 10 lung SCC samples and the results confirmed that *FZD3* is up-regulated in 7 out of 10 samples as shown in Figure 5. However, *DVL2* is only up-regulated in 3 out of 10 samples. When we applied the same concordance analysis onto these 10 samples, 9 samples showed reduced or unchanged expression of *CTNNB1*. Nearly half of these samples show that *FZD3 and DVL2* have the same pattern of expression. *FZD3* and *DVL2* are increased in 67% and 33% of the samples, respectively. These results are consistent to what was observed in the first panel of lung tumors of 58% and 41%, respectively. Limited knowledge exists of the involvement of *FZD3 and DVL2* in cancer. *FZD3* is reported to be down-regulated in ovarian cancer (Tapper et al. 2001) but up-regulated in chronic lymphocytic leukemia (Lu et al. 2004). Although *DVL2* has never been directly linked to cancer, its associations with Rho GTPases have been reported. Rho family of proteins are involved in a number of essential cellular processes such as cell growth, lipid metabolism, cytoskeleton architecture, membrane trafficking, transcriptional regulation, and apoptosis (Aznar and Lacal, 2001), with many of those processes disrupted in cancer.

Lastly, the *LRP5* (of the canonical pathway) and *SFRP4* pair is concordant in 71% of the samples with a corresponding correlation coefficient of 0.49 (*p* = 0.04). Interestingly, relationships between LRPs and sFRPs have not been previously reported. A total of 6 out of the 17 samples show coordinate down-regulation of *LRP5* and *SFRP4* in lung tumors. *LRP5* is a single transmembrane co-receptor that forms an active complex with the Fzd protein and an incoming Wnt ligand, to activate the canonical Wnt signaling pathway. As for *SFRP4*, although this protein exhibits the same domain architecture as other sFRP family members, its expression behaviour is different from its other family members. In contrast to the other sFRP members, *SFRP4* has been shown to be up-regulated where there is positive expression of *CTNNB1* (Feng Han et al. 2006) in a study involving human colorectal carcinoma. *In vitro* studies have also shown that overexpression of *SFRP4* does not lead to reduced expression of *CTNNB1* (Suzuki et al. 2004). Although the mechanisms behind the activation of the canonical pathway by *sFRP4* in these studies still needs more investigation, past and present evidence suggests that the sFRP genes may have more complex roles in addition to their pre-defined roles as Wnt antagonists.

## Conclusions

Based on the results in this study, the non-canonical pathway is active in normal lung. Activation of the non-canonical pathway in development has been associated with the control of specific morphogenetic movements during and following vertebrate gastrulation. This is one of the first reports to show activity of the non-canonical pathway in the human adult lung at the gene expression level. Previous studies of lung tumors have mainly focused on the canonical components. However, tumor gene expression analysis in this study shows that in fact, the non-canonical pathway may provide an alternative explanation to the proliferation of lung cancer cells. Further investigation at the protein level and phosphorylation state of CTNNB1 will provide a more comprehensive understanding of the biological impact of changes in the non-canonical components. We suggest that the non-canonical pathway may have a more prominent role in lung cancer than previously reported and future studies of the WNT pathway should encompass both the canonical and the non-canonical branches.

## Supplement Material

**Table S1:** Primer sequences and conditions for RT-PCR analysis.

Gene name	Primer sequence	MgCl _2_ (mM)	Cycles	Tm (°C)
DVL2	5′-aatcccagcgagttctttgt-3′ 5′-caatctcctgtatggcagca-3′	1	35	58.3
FZD1	5′-tacacgaggctcaccaacag-3′ 5′-gagcctgcgaaagagagttg-3′	1	35	52.3
FZD2	5′-catcgaggccaactctcagt-3′ 5′-gtgccgatgaacaggtacac-3′	1.5	35	52
FZD3	5′-tgagtgttcgaagctcatgg-3′ 5′-ttaactctcggggacaccaa-3′	1.5	30	60.9
FZD6	5′-caggcaggcagtgtatctga-3′ 5′-accacctccctgctcttttc-3′	2	30	58
LRP5	5′-cccgtcacaggtacatgtact-3′ 5′-gaacgagccgtccaggtt-3′	1	30	55
LRP6	5′-ttccaggaatgtctcgaggt-3′ 5′-ggttcaaaattgcagggaag-3′	1	35	51
SFRP1	5′-gagctccagtttgcatttgg-3′ 5′-tagggtgctctcctcaaaca-3′	1	35	58
SFRP2	5′-gacctgaagaaatcggtgct-3′ 5′-atgcgcttgaactctctctg-3′	1	35	60
SFRP3	5′-tgttaccagagcctctttgc-3′ 5′-gagaatgcccaaaaggcata-3′	2	35	64
SFRP4	5′-gtttccaaagcggagacttc-3′ 5′-atggcttgtgatggcttaca-3′	2	35	62.1
SFRP5	5′-actggagggtgttttcacga-3′ 5′-ctcccctgcctactttctga-3′	2	35	63.4
WNT1	5′-acagagccacgagtttggat-3′ 5′-gaggcaaacgcatctttgag-3′	1	35	55
WNT3A	5′-agagctgctggtctcatttg-3′ 5′-aggaaagcggaccatttctc-3′	2	35	58
WNT5A	5′-tggaccatgtgtggtgtctc-3′ 5′-gtgcagcactgtccagattt-3′	2	35	60.9
WNT11	5′-gaagccaccaggaacagaag-3′ 5′-gccctgaaaggtcaagtctg-3′	2	31	64
CADH	5′-agccatgggcccttggag-3′ 5′-ccagaggctctgtgcaccttc-3′	1	40	50
VIM	5′-tggcacgtcttgaccttgaa-3′ 5′-ggtcatcgtgatgctgagaa-3′	1	35	55
CTNNB1	5′-gagcctgccatctgtgctct-3′ 5′-acgcaaaggtgcatgatttg-3′	1	35	60

Figure S1 Pairwise expression profile analysis (tumor versus matched normal) of non-canonical and canonical Wnt pathway components in 20 SCC samples. Each tumor and normal pair is represented as an individual case, numbered from Case 1 to Case 20. For each gene, color gradient shading represents magnitude of over and underexpression.

## Figures and Tables

**Table 1:** Pairwise expression correlation of genes in WNT pathway.

Gene Pairs		(%)	R	*p*val
*Wnt1*	*Wnt11*	53	0.22	0.39
*B-catenin*	*sFRP5*	53	0.04	0.87
*B-catenin*	*Wnt3a*	59	0.14	0.59
*B-catenin*	*Lrp6*	53	0.41	0.11
*sFRP5*	*Wnt3a*	53	−0.02	0.95
*sFRP5*	*Lrp6*	53	0.35	0.17
*sFRP5*	*sFRP4*	53	0.31	0.22
*Wnt3a*	*sFRP1*	59	0.06	0.81
*Wnt3a*	*sFRP4*	59	0.45	0.07
*Fzd1*	*Lrp5*	53	0.48	0.05
*Fzd1*	*sFRP4*	53	0.45	0.07
*Fzd3*	*sFRP2*	53	0.3	0.24
*Fzd3*	*Dvl2*	77*	0.6	0.01
*Lrp5*	*sFRP4*	71*	0.49	0.04
*sFRP1*	*sFRP4*	59	0.49	0.04
*sFRP1*	*Wnt5a*	59	0.67	0
*sFRP2*	*Wnt5a*	59	0.69	0
*sFRP2*	*Dvl2*	53	0.05	0.86
*sFRP2*	*Fzd6*	53	0.31	0.22
*sFRP2*	*Fzd2*	53	0.46	0.07
*sFRP3*	*Wnt11*	59	0.28	0.28
*sFRP4*	*Wnt5a*	59	0.78	0
*Wnt5a*	*Fzd6*	53	0.55	0.02
*Wnt5a*	*Fzd2*	65*	0.7	0
*Wnt5a*	*Wnt11*	53	0.48	0.05
*Fzd6*	*Fzd2*	53	0.48	0.05
*Fzd2*	*Wnt11*	53	0.43	0.08

* denote gene pairs that are over 65% similar in the 17 samples

**Abbrevations:** R:Spearman correlation coefficient; *p*val:p-value of spearman correlation coefficient.
