# Supplementary figures and images for: Disruption of the Non-Canonical WNT Pathway in Lung Squamous Cell Carcinoma
Source: Clin Med Oncol. 2008 Apr 1;2:169–79. (PMC2855195)

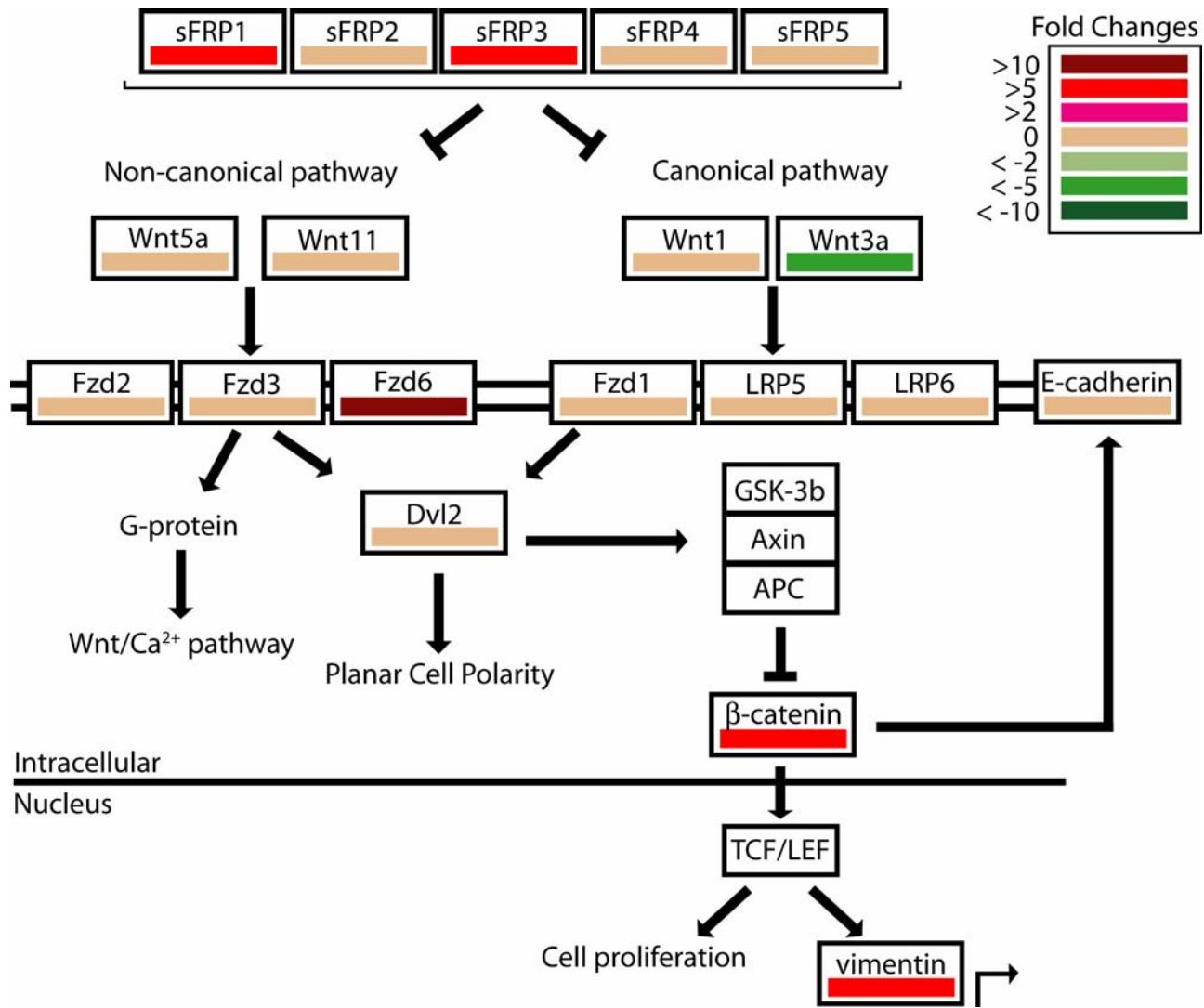

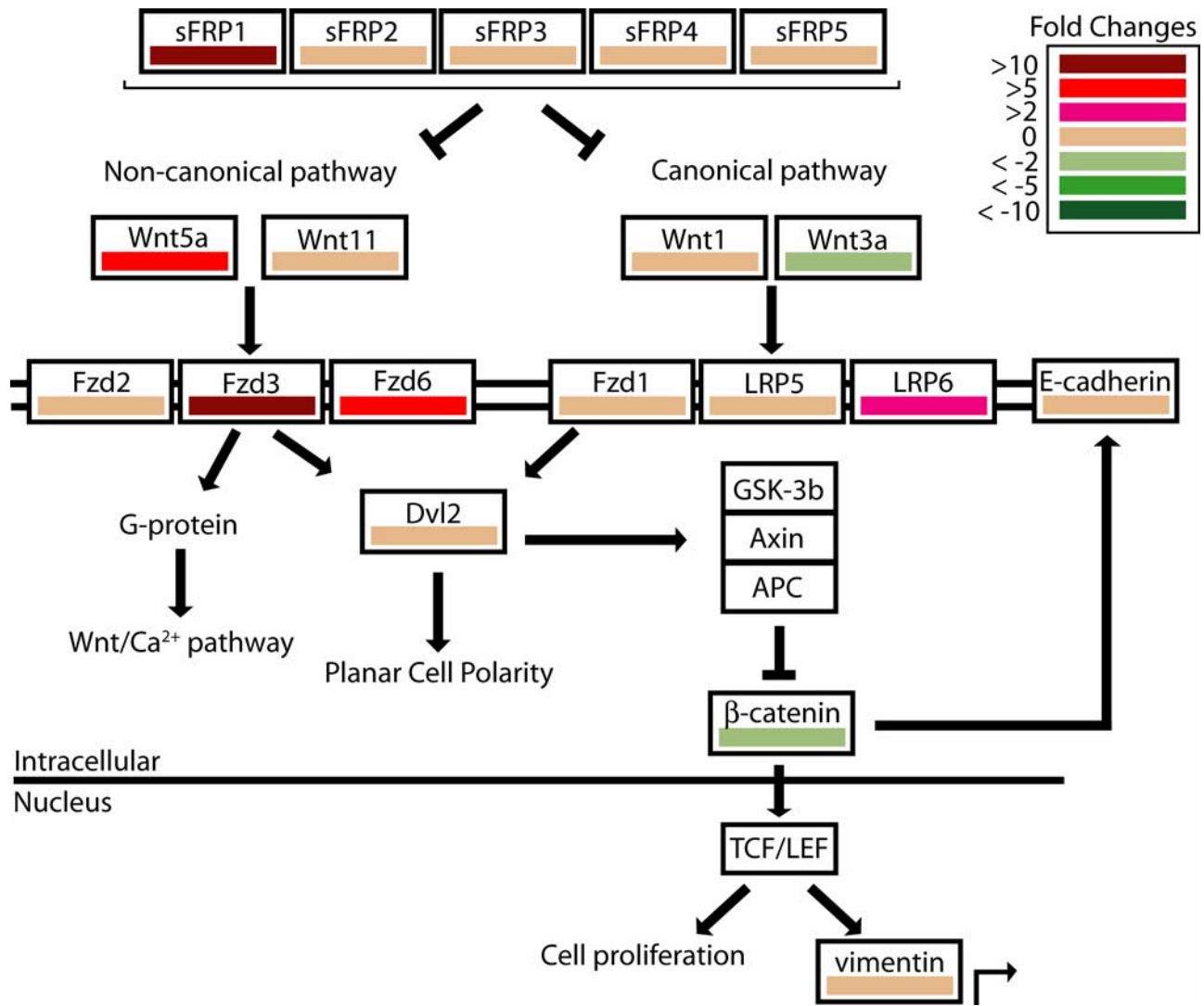

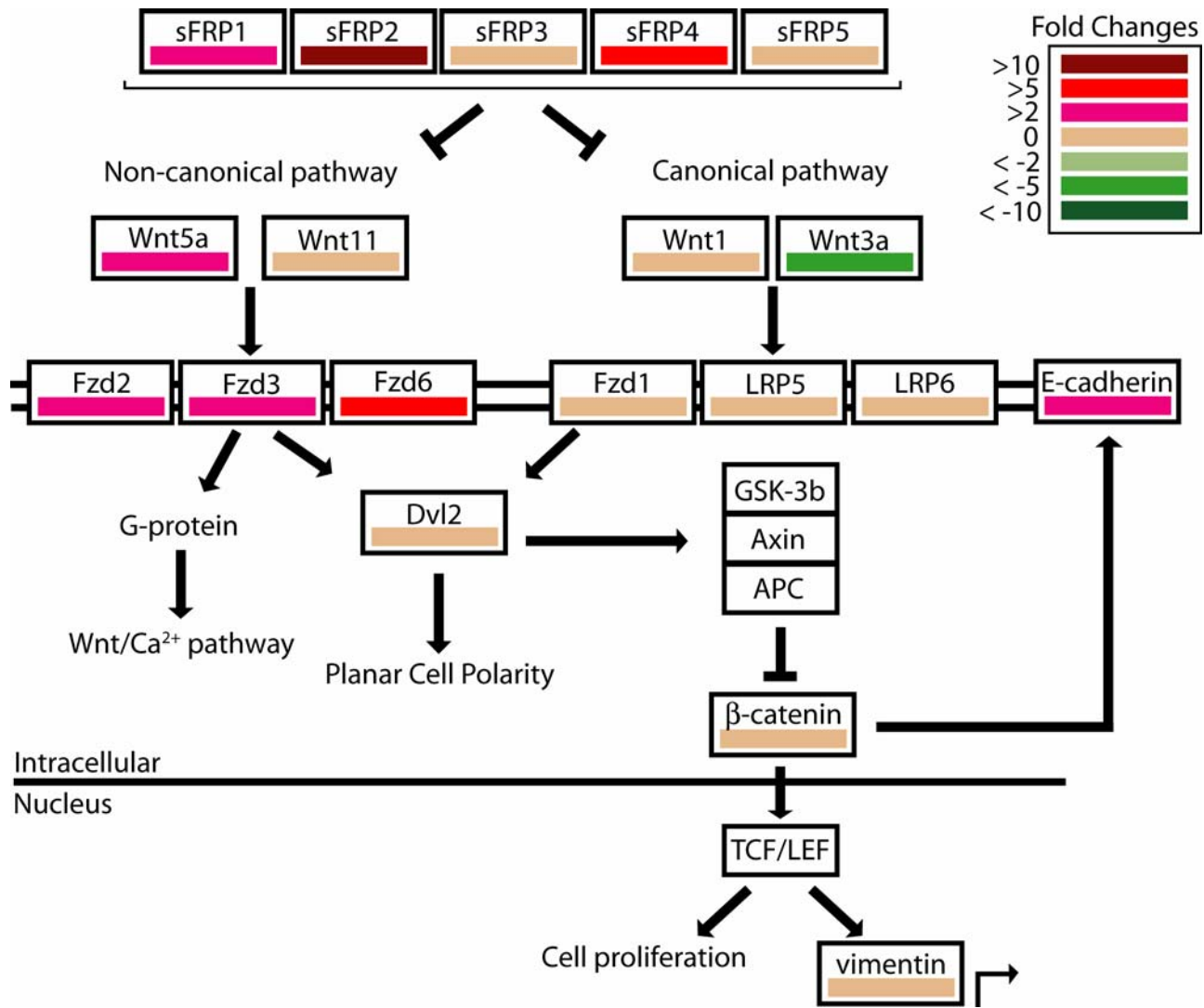

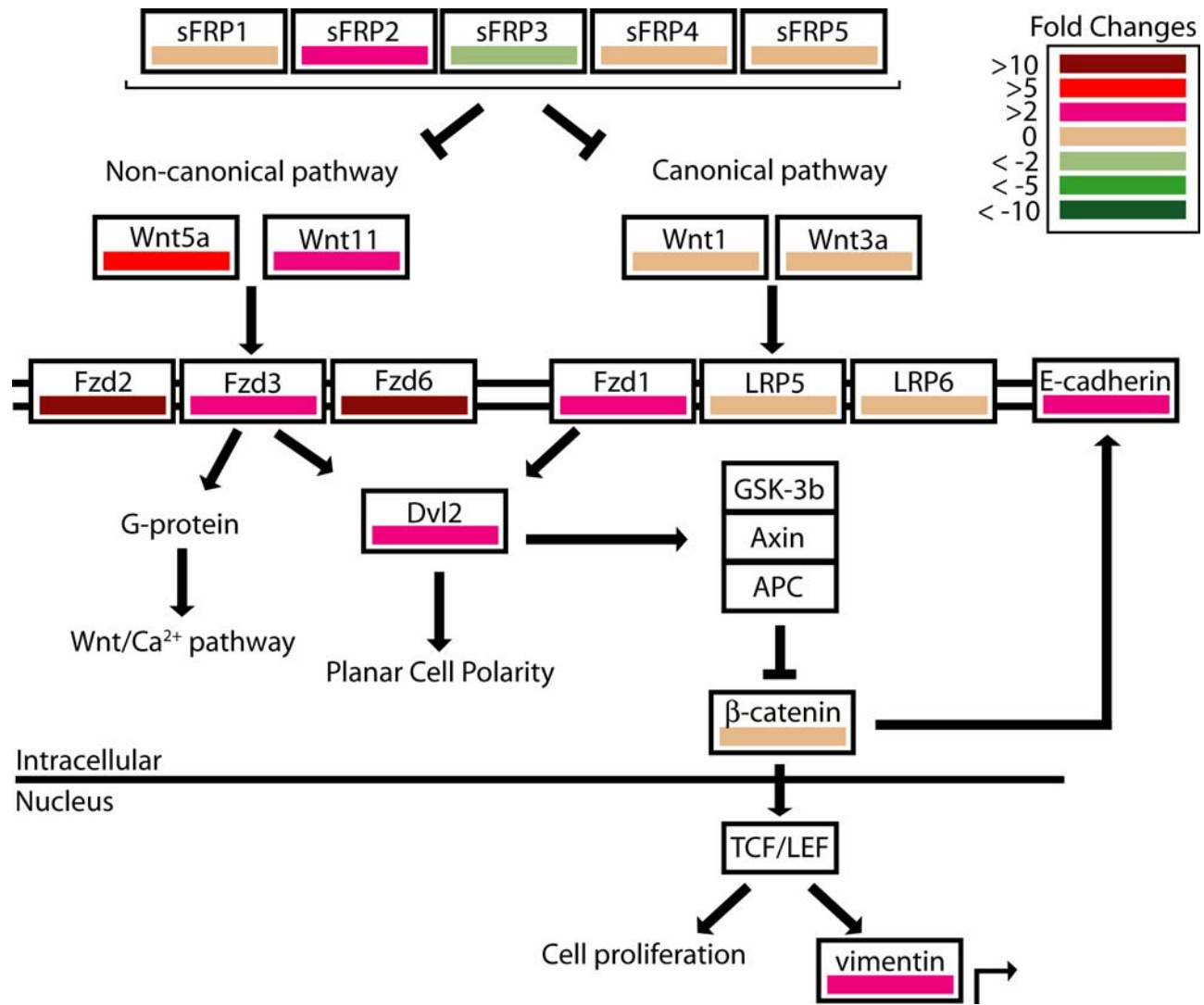

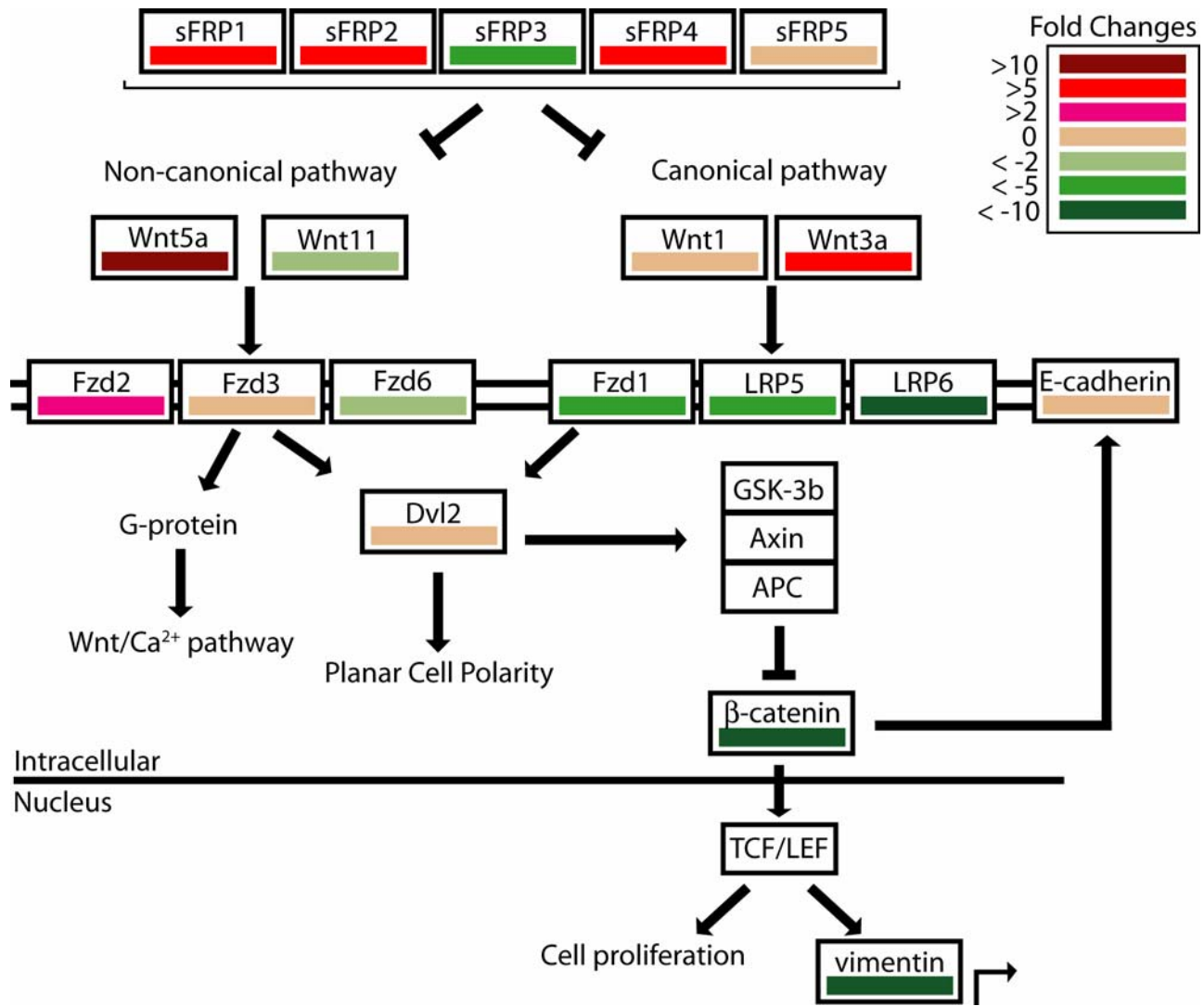

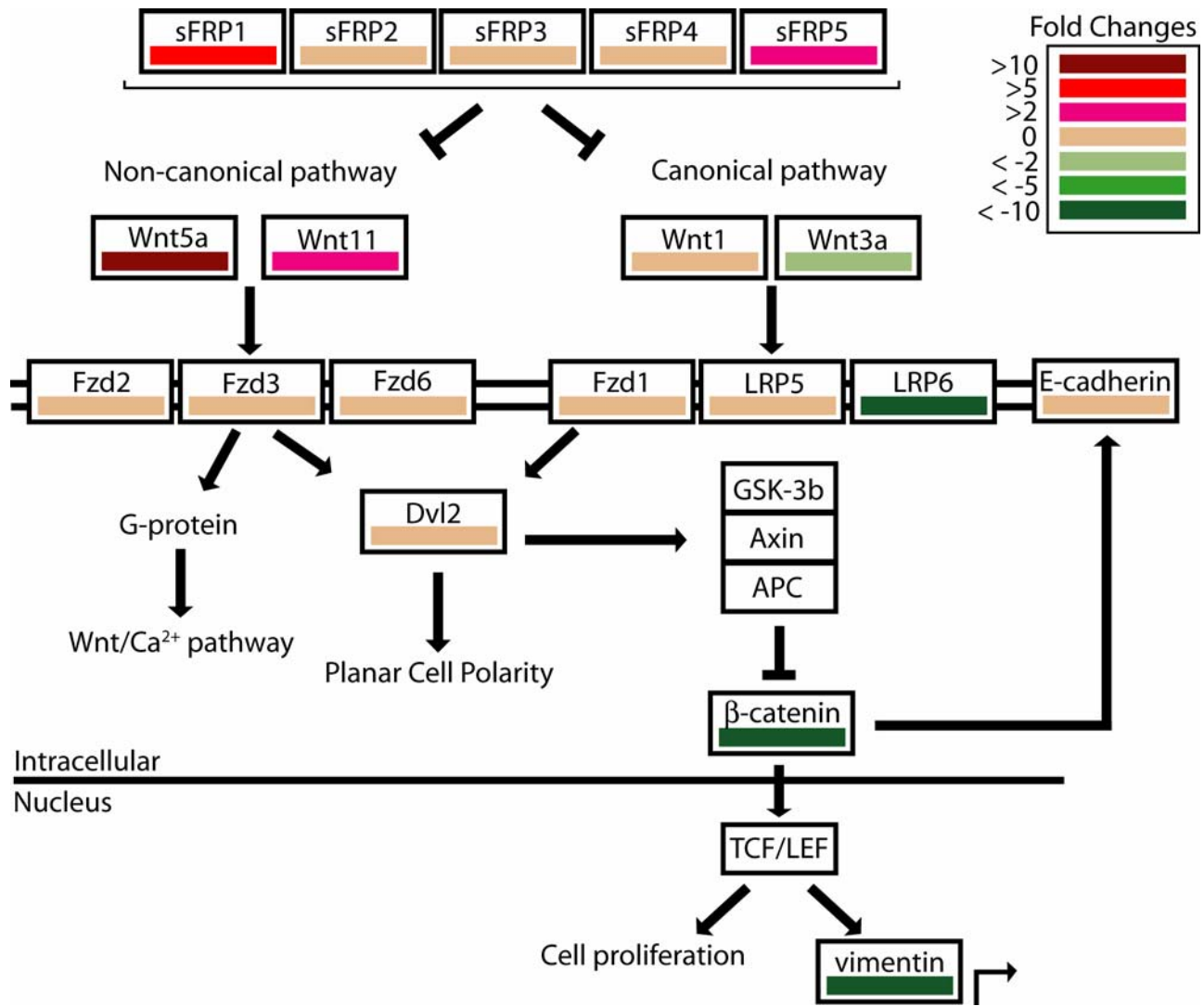

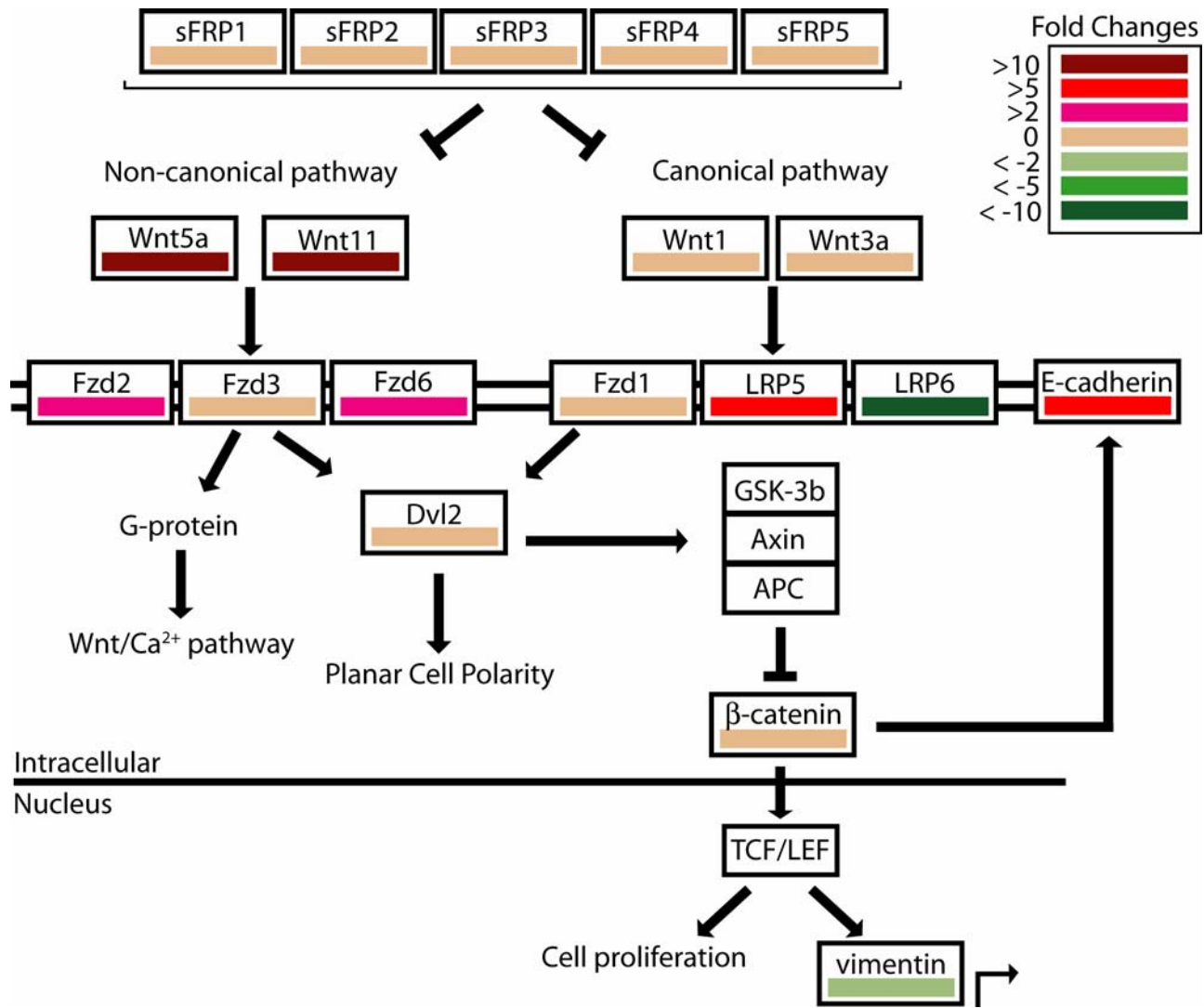

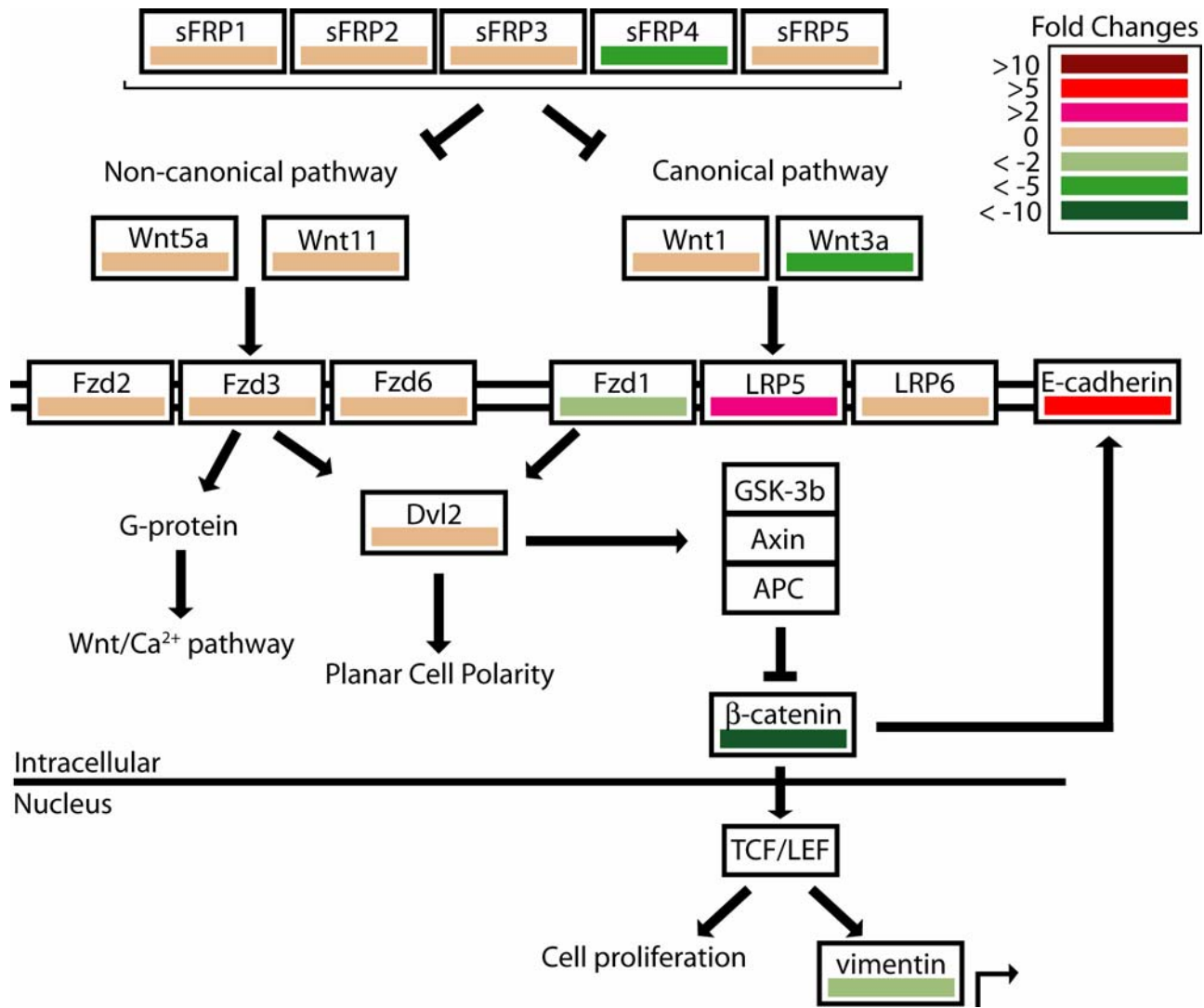

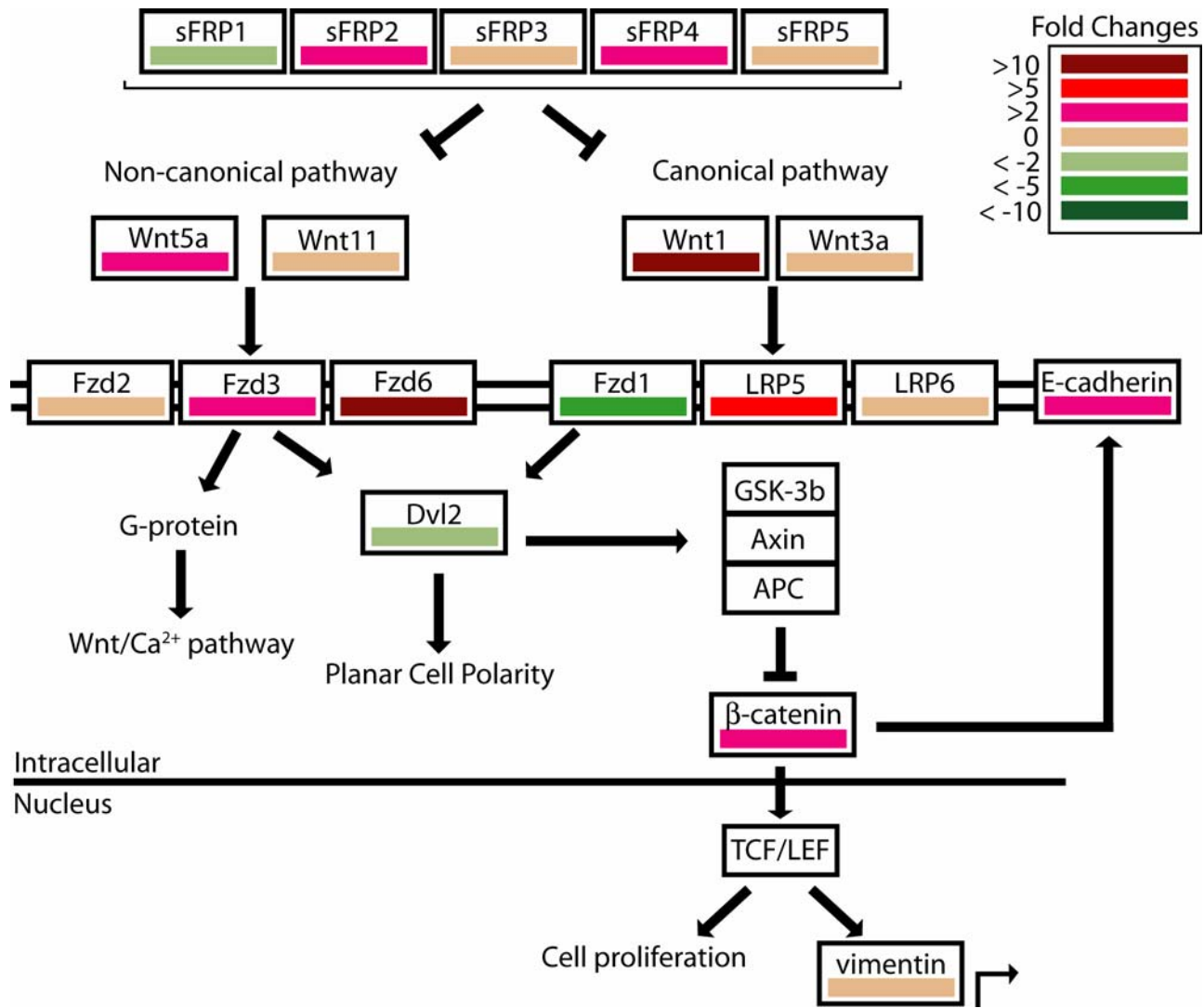

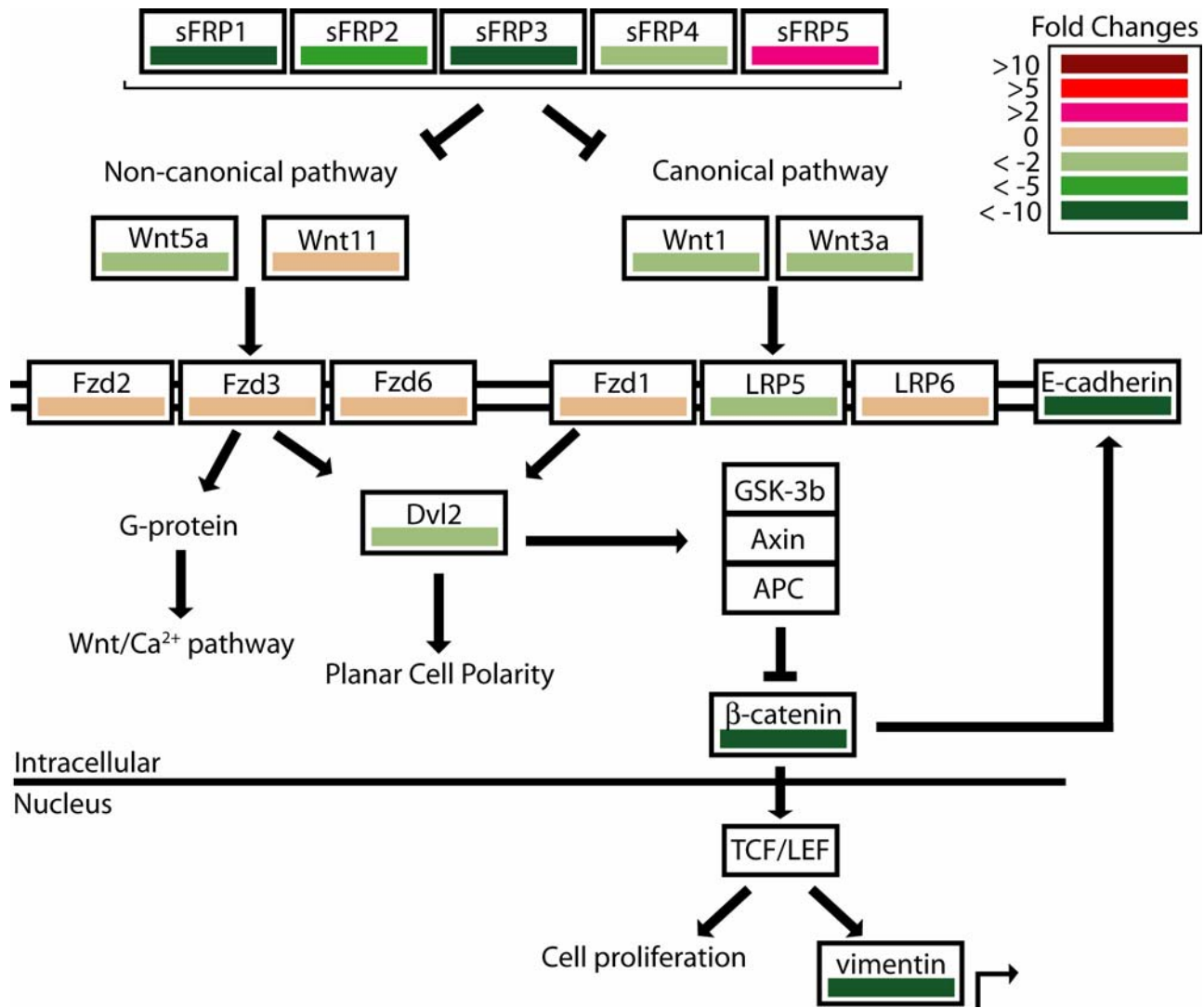

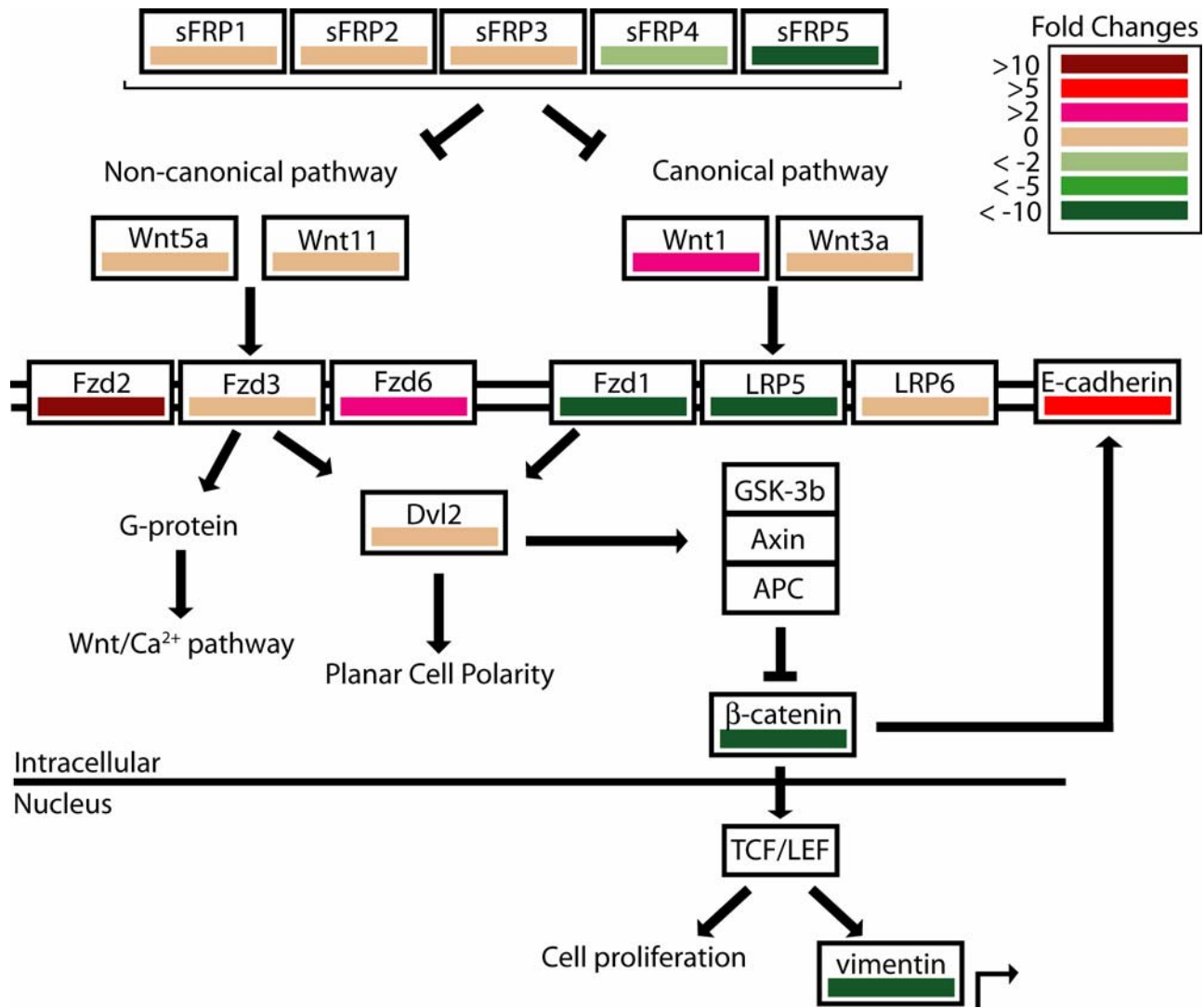

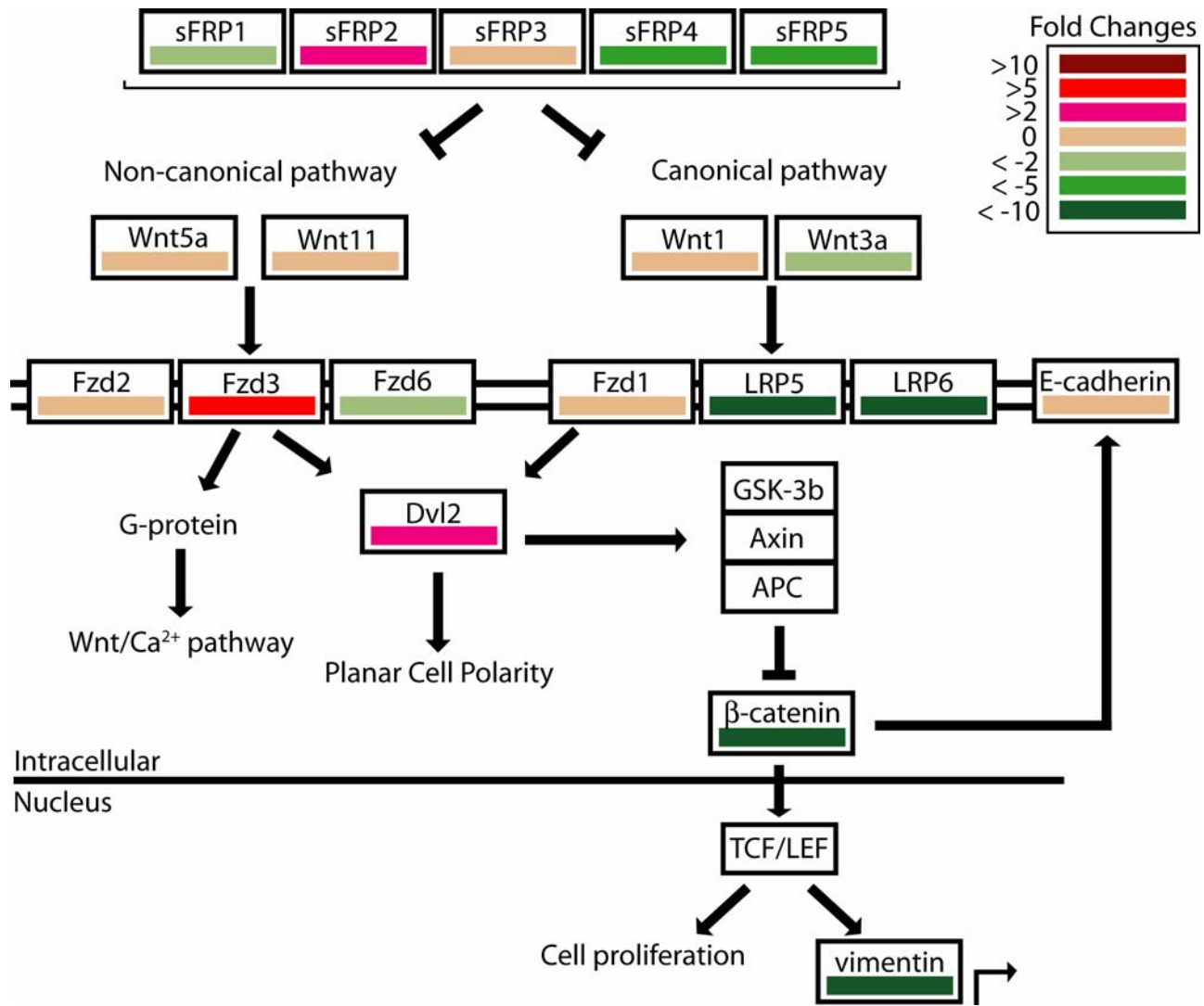

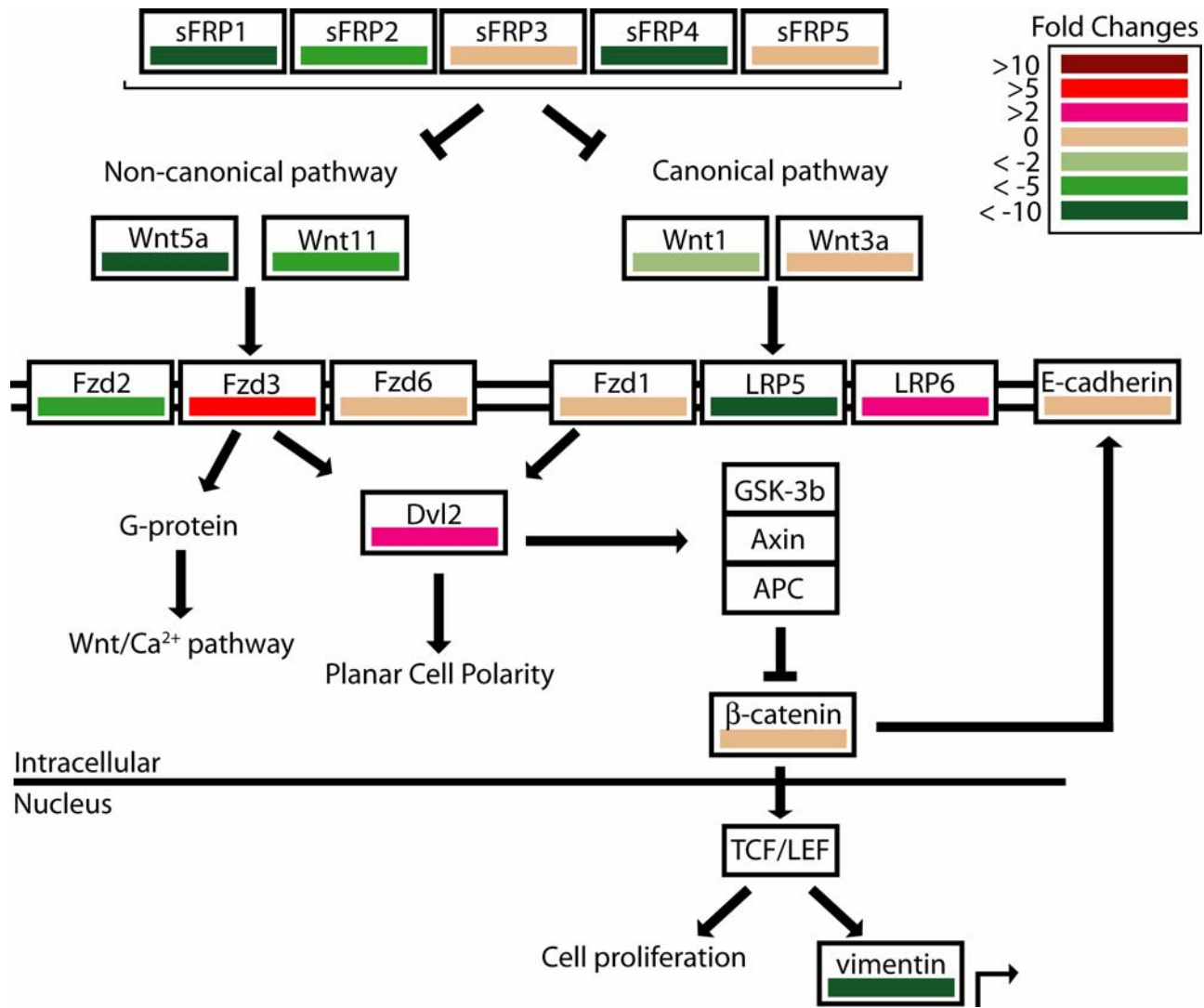

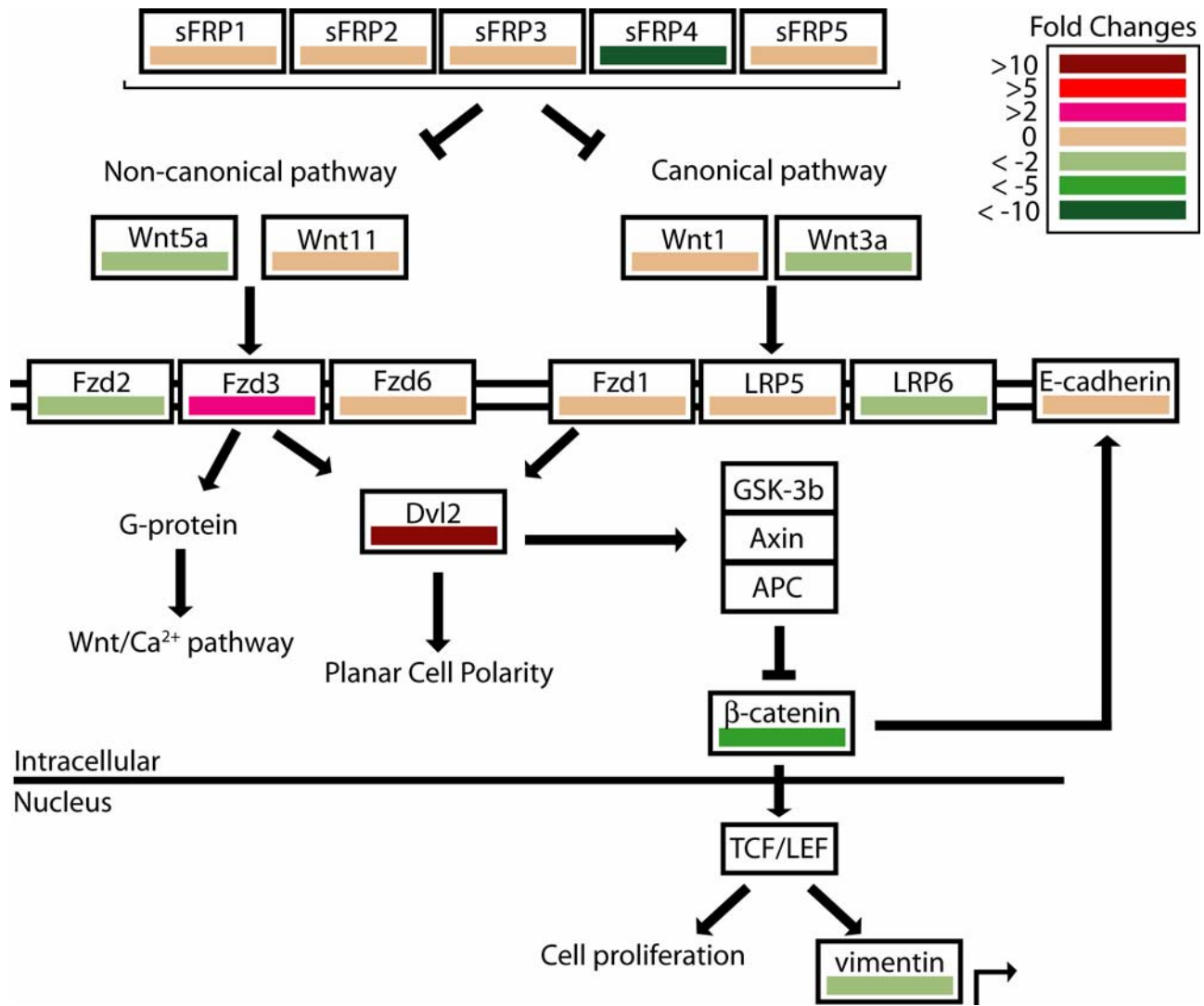

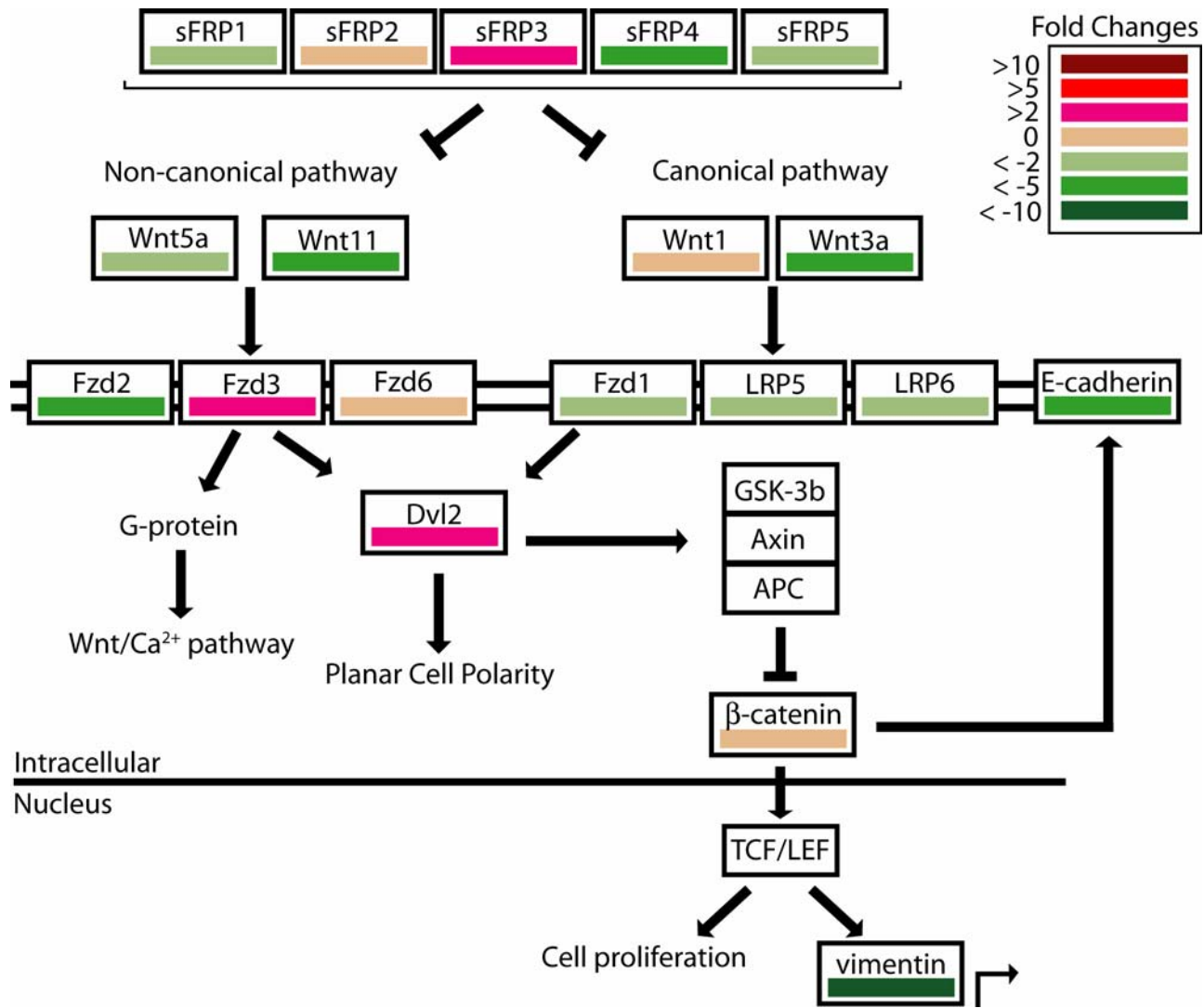

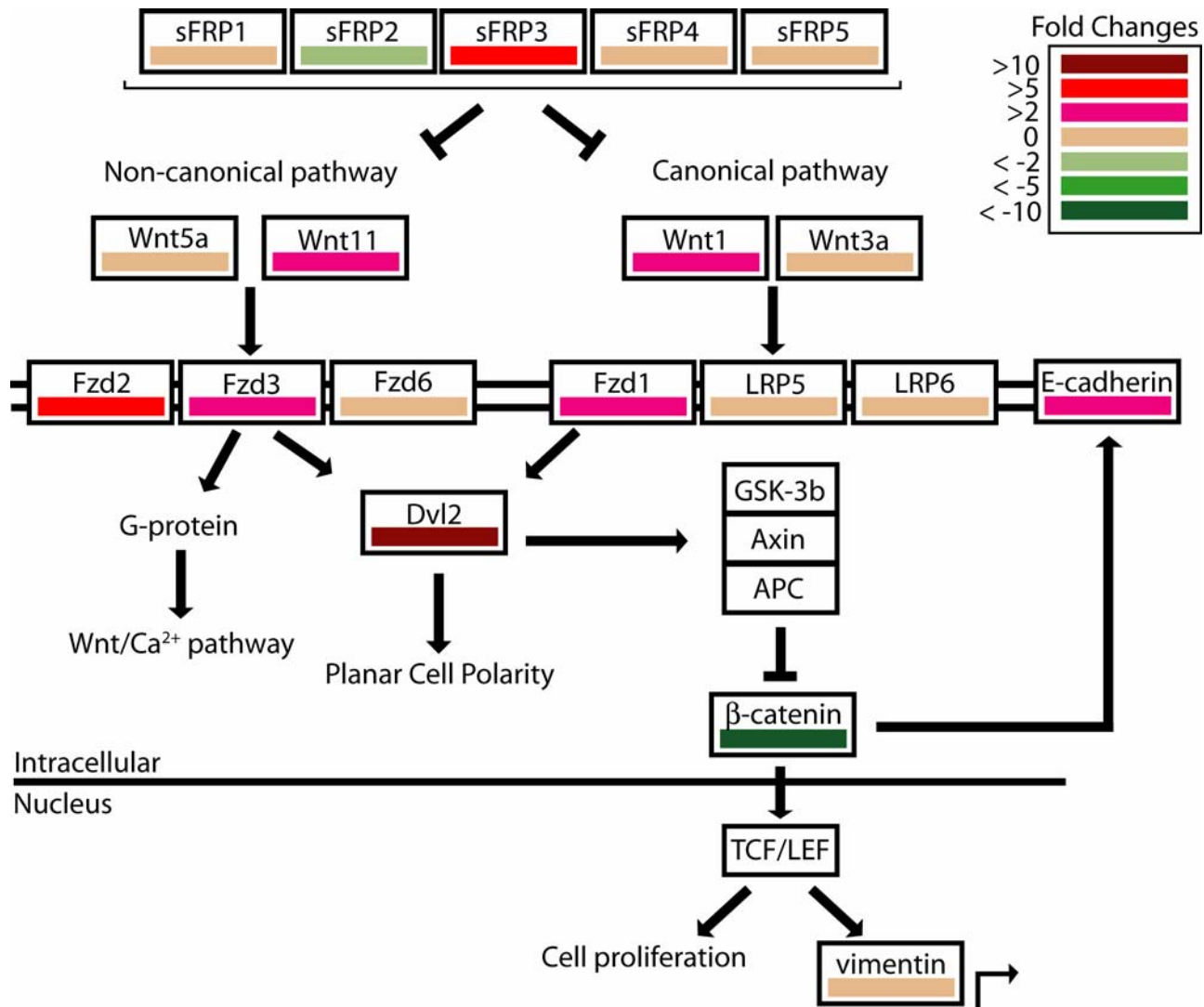

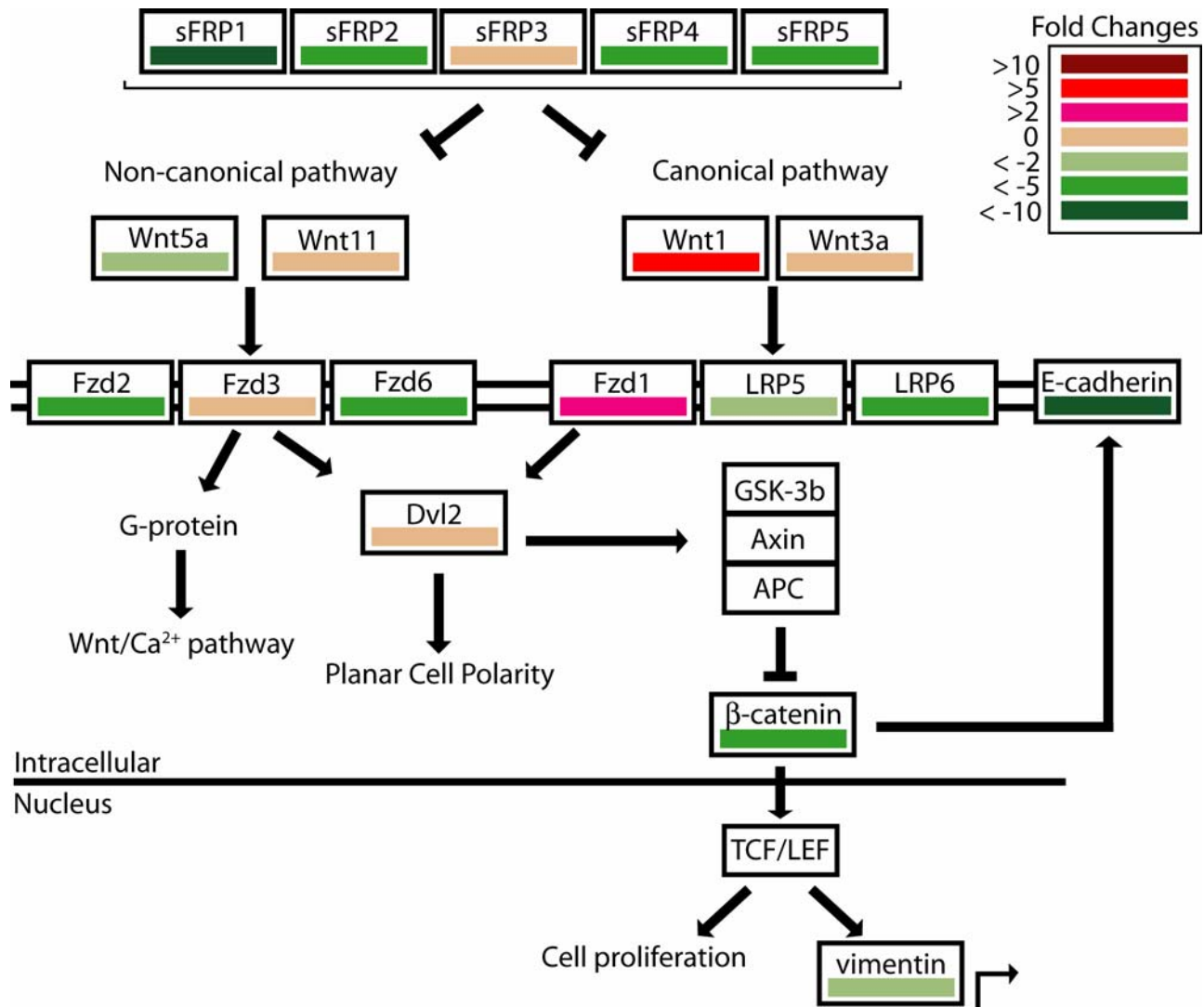

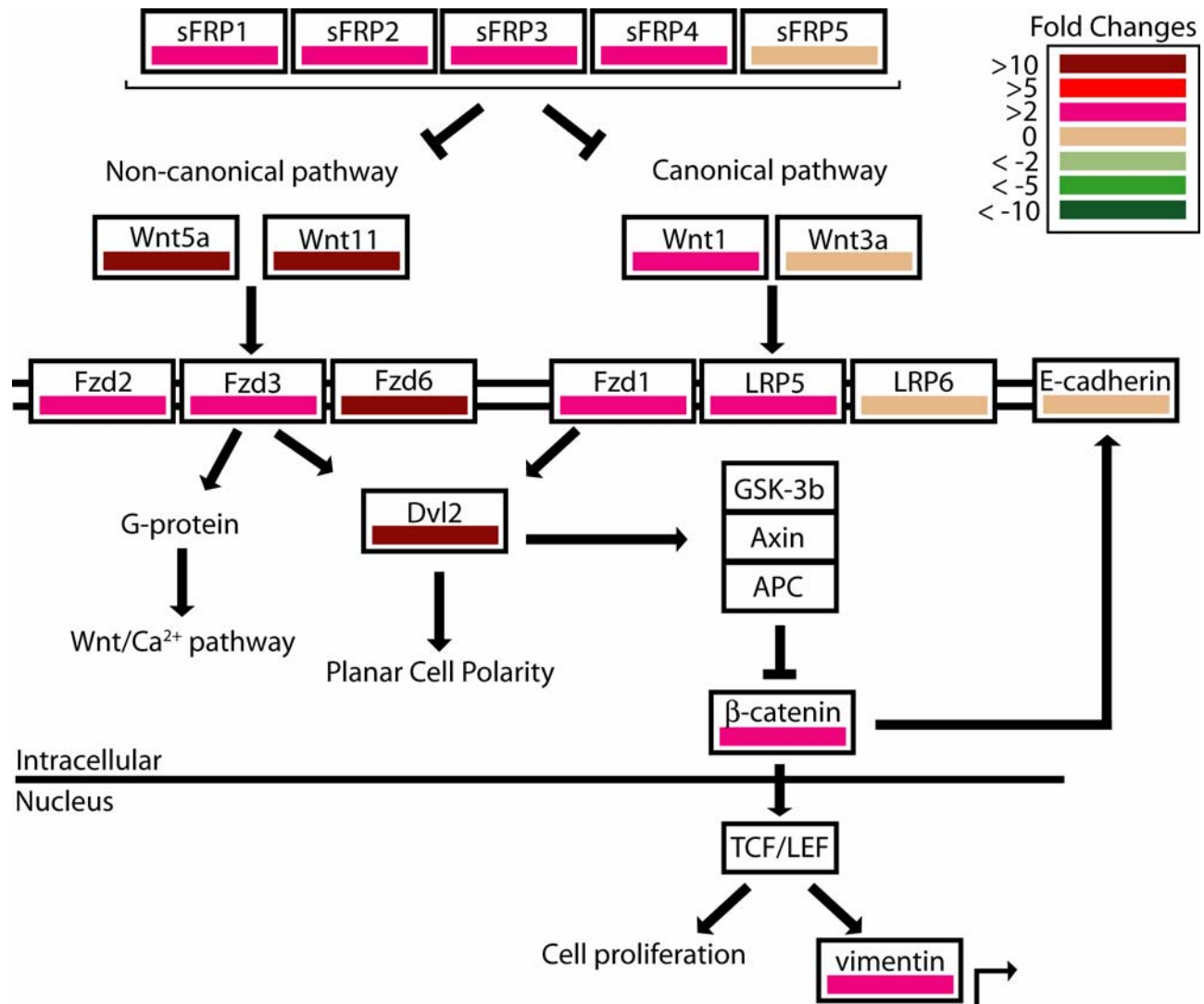

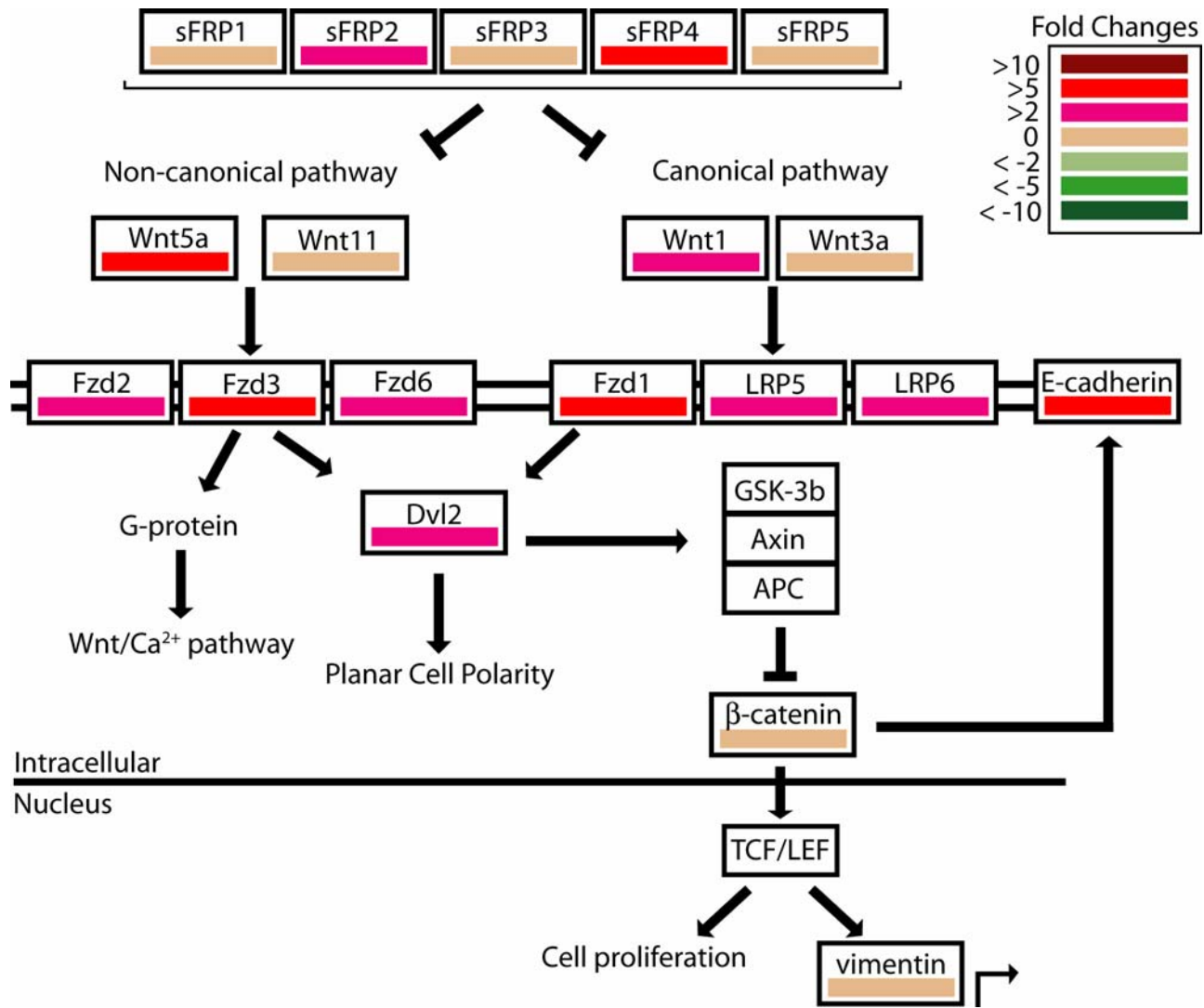

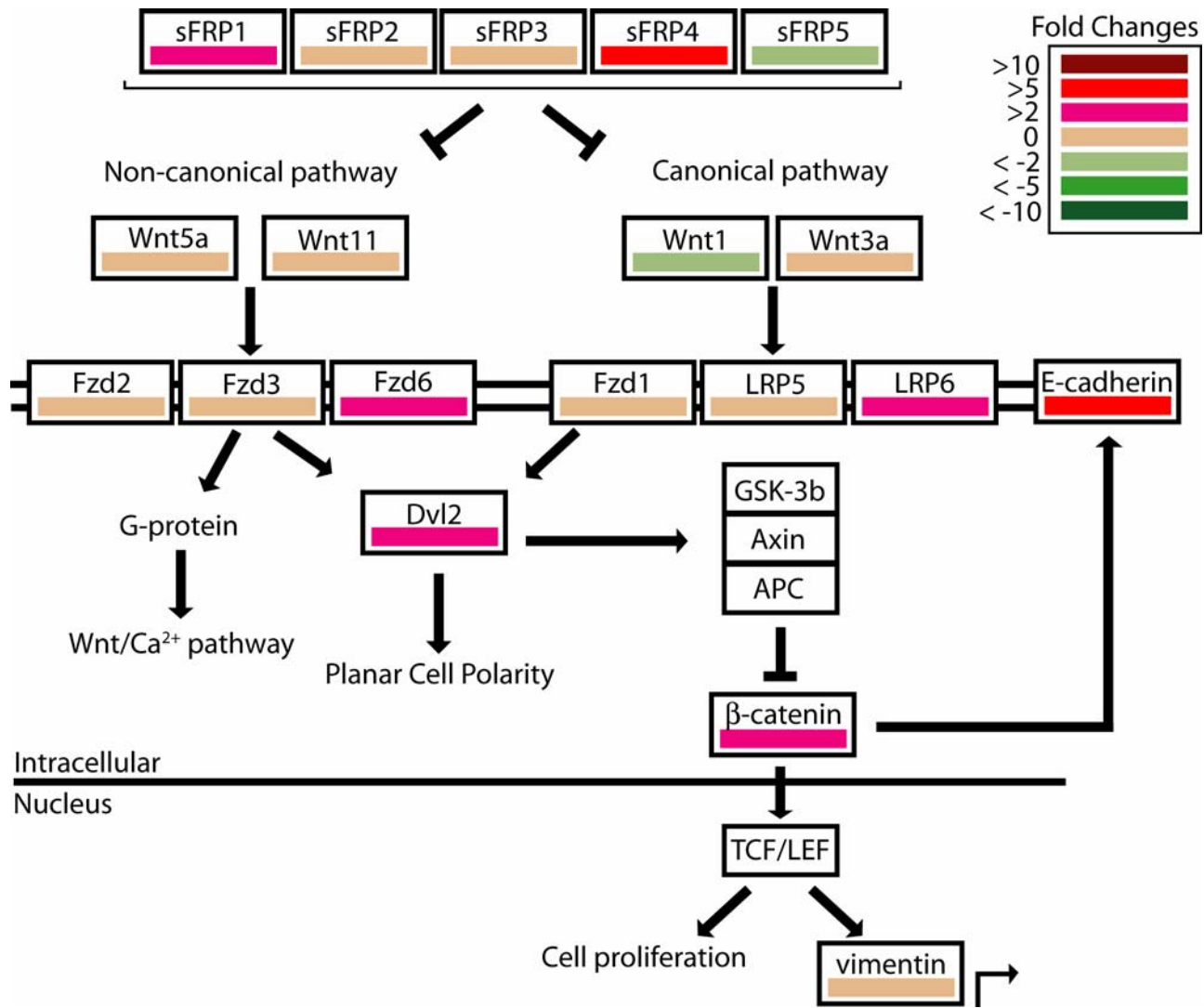

Supplement: Figure S1 — Pairwise expression profile analysis (tumor versus matched normal) of non-canonical and canonical Wnt pathway components in 20 SCC samples. Each tumor and normal pair is represented as an individual case, numbered from Case 1 to Case 20. For each gene, color gradient shading represents magnitude of over and underexpression. [file SupplFigures.pdf]
